# Skin Grafting

**Published:** 1871-01

**Authors:** 


### SKIN GRAFTING.

This operation, it would appear, from our recent journals, has been pretty frequently resorted to, of late, in the London hospitals.

Mr. Mason has performed it at the Westminster Hospital in nine instances, as is stated in the *Lancet* of October 22, 1870.

The first was that of a girl, with a cicatrix of a burn in the neck, of which a separate and detailed account will be given on another occasion.

The second was that of a woman who, for three years, had an ulcer on the leg, measuring about four inches by three. Three pieces of skin, of the size of a canary seed, were snipped from the front of the upper arm, and simply placed on the ulcer, and retained in position by means of a strip of transparent plaster, made by Ewen, of Jermyn street, and over this, water dressing and a bandage were applied. At the end of a month the ulcer had nearly healed, each of these pieces having, in a fortnight, attained the size of a fourpenny piece.

The third case was that of a man, with a flabby-looking ulcer, as large as the hand, situated in the groin. Four small pieces, from the front of the upper arm, were grafted. Three failed to grow, and the fourth, after one month, was only the size of a pea.

The fourth subject was a woman, with an unhealthy ulcer of the leg, extending nearly all around the limb. Four pieces were grafted, and they all failed to grow.

The fifth, a woman, with an ulcer of the leg, of four years' standing, and two by three inches in size. Two pieces of skin were grafted, and, in three weeks, each measured a quarter of an inch in diameter.

The sixth, a man of middle age, with an ulcer of the leg, four by three inches in size, of nearly four years' standing, which was sloughing at the time of admission. Charcoal and linseed poultices were first applied, and the wound soon showed fairly healthy granulations, on which four pieces were grafted; and on the strips being removed, four days later, they were all found to have adhered. When seen eleven days after the operation, they were spreading rapidly.

The seventh, a girl, aged twenty, with a flabby ulcer on thigh, of eight months' standing. Two pieces were grafted with good result.

In the eighth and ninth cases there were smaller ulcers, in which one piece only was grafted. They rapidly recovered.

Mr. Mason, without committing himself to a decided opinion on this interesting operation, believes that its success depends on the ulcer being, if not in a healing condition, at least of a healthy character. He had understood from Mr. Pollock, who first showed him the operation, that the grafted portions often disappeared for a time, and were, as it were, reproduced in the course of a few days. Mr. Mason has never noticed this phenomenon. In his successful cases, the grafted pieces have, on the contrary, never faded away, or diminished, but remained centres of centrifugally spreading growth. They have, in some instances, assumed, in the course of a few days, a cupped or depressed appearance, as if a kind of very superficial ulceration were proceeding; but they have ultimately filled up again, and the process of healing has proceeded. The pieces of skin have only disappeared in cases where they have perished. The situations most favorable for this procedure are, Mr. Mason thinks, those where—as in the case of ulcers of the leg—pressure can be readily applied, so as to keep the grafted skin permanently fixed for three or four days.

Perhaps the difference between the results of Mr. Mason's experiments and those of other operators, as to the disappearance of the transplanted skin, may be accounted for by the rather large size of the portions (picked up in an ordinary dressing forceps) which he cuts off.—*Medical News.*

